# PF-AGCN: an adaptive graph convolutional network for protein–protein interaction-based function prediction

**DOI:** 10.1093/bioinformatics/btaf473

**Published:** 2025-08-26

**Authors:** Shumin Yang, Yuhan Su, Yuchen Lin, Qin Lin, Zhong Chen

**Affiliations:** School of Electronic Science and Engineering, Xiamen University, Fujian 361005, China; School of Electronic Science and Engineering, Xiamen University, Fujian 361005, China; School of Electronic Science and Engineering, Xiamen University, Fujian 361005, China; First Affiliated Hospital of Xiamen University, Fujian 361000, China; School of Electronic Science and Engineering, Xiamen University, Fujian 361005, China

## Abstract

**Motivation:**

Proteins carry out most biological processes via interactions with other proteins, known as protein–protein interactions (PPIs). Accurately predicting PPIs is crucial for understanding protein function, yet existing methods often fall short in capturing their complex and hierarchical nature.

**Results:**

We propose PF-AGCN, an adaptive graph convolutional network that leverages two distinct graph structures: a function graph representing hierarchical Gene Ontology term relationships and a protein graph modeling direct interactions between proteins. Unlike traditional graph attention networks, PF-AGCN preserves the original biological structures while dynamically learning new relationships, ensuring the retention of essential biological information. Additionally, our framework integrates a protein language model with stacked dilated causal convolutional neural networks, enabling the synergistic fusion of global sequence semantics and local structural patterns. Extensive experiments on a comprehensive protein dataset across three evaluation facets demonstrate PF-AGCN’s superior prediction accuracy.

**Availability and implementation:**

The source code is publicly available at https://github.com/smyang107/PFAGCN.

## 1 Introduction

### 1.1 Background

Proteins are fundamental to nearly all biological processes, and accurately predicting their molecular functions is essential for understanding complex cellular systems. Functional annotation is commonly facilitated by classification schemes such as Enzyme Commission numbers, Gene Ontology (GO), and Pfam, which categorize proteins based on enzymatic activity, biological processes, and structural domains, respectively (Alborzi *et al.* 2017). Given that proteins often operate in concert, analyzing protein–protein interactions (PPIs) is critical for precise functional inference (Jha *et al.* 2022). While experimental approaches such as yeast two-hybrid screening and mass spectrometry can identify PPIs, their limited scalability due to high cost and low throughput highlights the pressing need for computational alternatives (Walzthoeni *et al.* 2013).

Traditional computational strategies, such as sequence alignment, structural comparison, motif analysis, and classical machine learning (ML), have laid the groundwork for protein function prediction (Su *et al.* 2024). For example, tools like Basic Local Alignment Search Tool (BLAST) infer function through sequence homology but are limited to closely related proteins (Cozzetto *et al.* 2016). Structure-based methods rely on 3D conformational similarities but are constrained by the limited availability of experimentally resolved protein structures (Gherardini and Helmer-Citterich 2008). Motif-based techniques effectively detect conserved regions yet often fail to capture broader contextual information. The third Critical Assessment of Functional Annotation (CAFA) highlighted the rising prominence of ML in functional annotation (Zhou *et al.* 2019); however, traditional models such as *K*-nearest neighbors (KNN), random forests, and support vector machines (SVM) depend heavily on manual feature engineering and often struggle to model complex sequence patterns (Bonetta and Valentino 2020).

Recent advances in deep learning (DL) have enabled end-to-end learning of hierarchical features directly from raw sequences and structures (Lv *et al.* 2019). Convolutional neural networks (CNNs), adapted from computer vision, excel at extracting local patterns in amino acid (AA) sequences, as demonstrated by models such as Proteinfer and DeepGO (Kulmanov *et al.* 2018). However, these models often neglect long-range dependencies, including those mediated by PPIs. Recurrent neural networks (RNNs), particularly long short-term memory (LSTM) networks, are more effective at capturing sequential dependencies, and hybrid CNN-RNN models further enhance performance by learning both spatial and temporal features (Yao *et al.* 2021). Pre-trained language models such as Evolutionary Scale Modeling (ESM) and SeqVec have advanced the field by providing robust sequence embeddings. ESM leverages transformers to model long-range dependencies, while SeqVec uses CharCNN-BiLSTM to encode both local and global patterns (Rives *et al.* 2021). Nonetheless, transformer models may struggle to capture fine-grained structural details due to attention diffusion (Fang *et al.* 2024). Recent innovations like topology-aware wavelet fusion networks mitigate this issue by incorporating dual-stream fusion mechanisms to enhance structural fidelity (Tian *et al.* 2024). In our work, we integrate the ESM model with stacked dilated causal CNNs to jointly capture both global and local sequence dependencies.

Graph convolutional networks (GCNs) have also gained traction for modeling protein structures and capturing PPIs, with approaches typically categorized as structure-based or PPI-based. Structure-based GCNs use residue-level graphs derived from contact maps or 3D conformational data—e.g. DNN+node2vec (Wang *et al.* 2023b), SkipGNN (Huang *et al.* 2020), and SEAL (Zhang and Chen 2018). Graph-based generative models further extend this by using graph transformers to design protein sequences from 3D structural graphs, exploiting spatial dependencies (Ingraham *et al.* 2019, Tang *et al.* 2023). Nonetheless, these approaches are hampered by the scarcity of resolved structures and high computational demands (Zhang *et al.* 2024). For instance, while the MAPE-PPI framework reduces computational overhead, it still faces challenges such as redundant operations during codebook learning and insufficient modeling of neighboring residue effects (Wu *et al.* 2024).

PPI-based GCNs represent proteins as nodes linked by interaction edges, often enriched with sequence features, as seen in models like BaPPI and DDMut-PPI (Tang *et al.* 2024, Zhou *et al.* 2024). Graph autoencoders have also been employed to fuse sequence and interaction topology for improved predictions (Yang *et al.* 2020). However, many models treat structural and interaction data independently, limiting their ability to capture the hierarchical and contextual complexity of protein functions, including GO relationships. Moreover, GCNs are prone to over-smoothing when stacked too deeply, which reduces their effectiveness on large-scale datasets (Wu *et al.* 2024). Capturing the intrinsic hierarchy of PPIs remains a key challenge, and few models effectively integrate both the internal structure of proteins and their external network context. The HIGH-PPI model addresses this by employing a hierarchical graph that incorporates both internal and external protein views, but it still struggles to fully utilize protein annotations and scale to large datasets (Gao *et al.* 2023). Recent advances have begun addressing these limitations by incorporating GO hierarchies as directed acyclic graphs (DAGs) (Liu *et al.* 2024) and applying self-attention to adaptively weight neighbor nodes (Lai and Xu 2022). While graph attention networks (GATs) improve feature aggregation, they may still compromise structural integrity by failing to preserve contact maps (Ieremie *et al.* 2022).

### 1.2 Novelty and contribution

Despite recent progress, DL-based protein function prediction still faces key challenges. CNNs capture local sequence patterns but miss global context like PPIs; RNNs model sequence order but ignore structural and interaction data; pre-trained transformers handle long-range dependencies yet suffer from attention diffusion, weakening local feature retention. GCNs integrate GO hierarchies and attention but face two limitations: (i) attention may distort original graph structures (e.g. contact maps), and (ii) PPIs are inadequately integrated with GO DAGs. To address these issues, we propose PF-AGCN, an adaptive GCN that fuses two complementary graphs: a “function graph” encoding GO hierarchies and a “protein graph” modeling structural contact maps. As GO terms cluster functionally similar proteins, the function graph also captures PPI-like associations. PF-AGCN employs an adaptive mechanism to preserve native graph structures while learning refined representations, enabling biologically consistent integration of structural and functional data. This unified framework enhances both interpretability and predictive accuracy by uncovering novel protein–function relationships. Our main contributions include:

- A dual-graph architecture that fuses GO-based functional hierarchies with protein interaction structures for holistic function prediction.
- An adaptive mechanism that maintains original graph topology while dynamically optimizing representations during training.
- A dual-branch module combining ESM and dilated causal CNNs to capture both global sequence semantics and local structural patterns.
- Superior performance on public benchmarks, validating the model’s effectiveness in integrating diverse biological information.

The rest of the article is structured as follows: Section 2 details the methodology and PF-AGCN architecture; Section 3 discusses the experimental setup and results; Section 4 concludes with final insights.

## 2 Methodology

**Figure 1.**
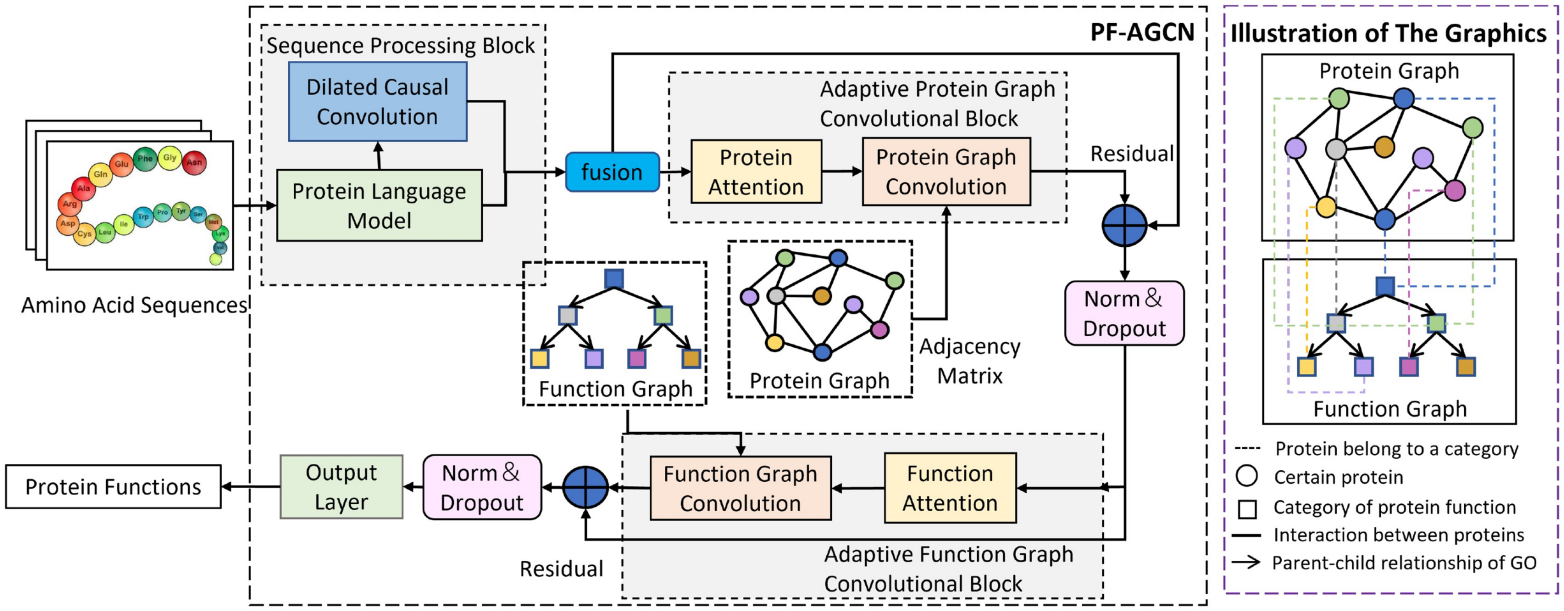
Illustration of the framework of the proposed PF-AGCN and the proposed graphics.

As shown in Fig. 1, the PF-AGCN framework comprises three key modules: the Sequence Processing Block (SPB), the Adaptive Protein Graph Convolutional Block (AP-GCB), and the Adaptive Function Graph Convolutional Block (AF-GCB). Central to the architecture is a novel protein functional attention mechanism that fuses protein- and function-level attention to capture functional correlations among proteins. The SPB combines a pre-trained transformer for global sequence modeling with stacked dilated causal CNNs for local structural feature extraction, producing comprehensive sequence embeddings. AP-GCB and AF-GCB apply attention-guided graph convolutions on protein and function graphs, respectively, to capture complementary PPI-related features. By embedding attention into graph convolution while preserving original topologies, PF-AGCN adaptively learns biologically meaningful representations, thereby enhancing protein function prediction.

### 2.1 Problem definition

Within the hierarchical structure of GO domains, we construct the corresponding functional hierarchy as a “function graph,” denoted by $G_{F}=(V_{F},E_{F})$, where $V_{F}$ is the set of function nodes indexed by $N_{F}=\{1,2,\ldots ,i,\ldots ,N_{F}\}$, and $E_{F}$ represents the set of directed edges capturing defined GO relationships. These hierarchical connections encode critical functional dependencies and semantic correlations among GO terms. The corresponding adjacency matrix, $A_{F}=[a_{F,i,i^{\prime }}]\in R^{N_{F}\times N_{F}}$, is derived from $G_{F}$, where $a_{F,i,i^{\prime }}=1$ indicates the existence of a directed edge from node $v_{F,i}$ to $v_{F,i^{\prime }}$, and 0 otherwise. As illustrated in Fig. 1, this “function graph” incorporates GO’s ontological structure as prior knowledge to enhance the modeling of functional relationships and improve the annotation of uncharacterized proteins.

Similarly, we define the “protein graph” as $G_{P}=(V_{P},E_{P})$, where $V_{P}$ denotes protein nodes indexed by $N_{P}=\{1,2,\ldots ,j,\ldots ,N_{P}\}$, and $E_{P}$ represents edges capturing known PPIs. The corresponding adjacency matrix $A_{P}=[a_{P,j,j^{\prime }}]\in R^{N_{P}\times N_{P}}$ is defined such that $a_{P,j,j^{\prime }}=1$ if an interaction exists between $v_{P,j}$ and $v_{P,j^{\prime }}$, and 0 otherwise. To enhance biological realism, known PPI data are incorporated as priors when constructing $A_{P}$.

Let $X\in R^{N_{P}\times U}$ denote the sequence information matrix, which includes AA sequences, ternary diagrams, and other relevant features, where *U* represents the embedding dimension of AA sequences. Simultaneously, $Y={\left[y_{i,j}\right]}^{N_{P}\times N_{F}}\in R^{N_{P}\times N_{F}}$ serves as the matrix indicating predicted protein functions. A binary notation is used, where $y_{i,j}=1$ signifies that the *j*th function is associated with the *i*th protein, while $y_{i,j}=0$ denotes its absence. To enhance protein function prediction, we formulate the task as a graph node classification problem, enabling the application of GCNs to effectively capture node representations within the network topology for function prediction. The task is formally defined as


(1) $$Y=M(X,G_{F},G_{P}),$$

where M(.) represents the proposed model for protein function prediction.

### 2.2 Sequence processing

To extract protein features and capture global dependencies, we employ the ESM-1b model to generate high-quality embeddings of protein sequences. ESM-1b is a 34-layer transformer-based architecture pre-trained on over 250 million sequences from the UniParc database, enabling it to learn biologically meaningful representations (Wang *et al.* 2023a). To accommodate input constraints, sequences exceeding 1024 AAs are truncated to the first 1000 residues. Each AA in the processed sequence is embedded into a 1280-dimensional vector, yielding a static representation $F_{\text{ESM}}$ that encodes rich sequence and evolutionary information. This embedding serves as the foundational input for downstream tasks such as protein function prediction.

**Figure 2.**
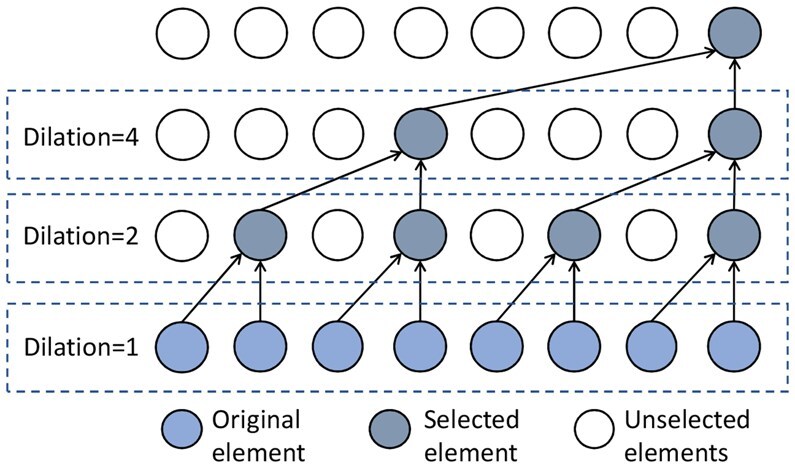
Illustration of dilated casual convolution with kernel size 2.

To complement global features with local context, we further refine $F_{\text{ESM}}$ using a hierarchical 1D dilated causal convolution (DCC) module. While the attention mechanism in ESM-1b excels at capturing long-range dependencies, it often underperforms in modeling short-range local motifs, such as hydrophobic clusters consisting of 3–5 residues (Rives *et al.* 2021). Dilated convolutions address this limitation by employing multi-scale receptive fields with dilation rates r=1,2,3,4, allowing the model to capture both fine-grained and broader local patterns. As illustrated in Fig. 2, we stack DCC layers to achieve exponentially growing receptive fields. Formally, let the protein embedding be represented as $D=[d_{0},d_{1},\ldots ,d_{N_{P}-1}]\in R^{N_{P}\times K}$, and let $v_{q}(s)\in R^{S_{q}}$ be a convolutional filter at layer *q*, applied at time step *t*. The DCC operation is defined as:


(2) $$F_{\text{DCC}}^{\left(q\right)}(t)=\sum_{s=0}^{S_{q}-1}v_{q}(s)\cdot d_{i}\left(t-r_{q}\cdot s\right)+b_{q},$$

where $r_{q}$ is the dilation factor and $b_{q}$ is a learnable bias specific to each channel. By increasing $r_{q}$ exponentially across layers q∈Q={1,2,…,Q}, with $r_{q}=2^{q-1}$, we obtain a receptive field of size $2^{Q}$, enabling efficient modeling of long-range contextual information. This design is particularly effective in tasks involving sequential data, such as natural language and protein sequences.

To aggregate the multi-scale features extracted from each DCC layer, we employ a gating mechanism (Wang *et al.* 2021):


(3) $$F_{\text{DCC}}=\sum_{q=1}^{Q}\alpha_{q}⊙F_{\text{DCC}}^{\left(q\right)},\;\alpha_{q}=\sigma \left(W_{q}[F_{\text{DCC}}^{\left(1\right)};\ldots ;F_{\text{DCC}}^{\left(4\right)}]\right),$$

where σ is the sigmoid function, $W_{q}$ is a learnable weight matrix, and ⊙ is channel-wise multiplication. When Q=4, the receptive fields span 3, 7, 15, and 31 residues, effectively capturing structural patterns such as beta-sheet folds (5–15 residues) and alpha-helices (10–20 residues). This integration of global embeddings with hierarchical local features enables robust and context-aware modeling of protein sequences.

To balance global and local feature contributions, we introduce a learnable gating mechanism with a trainable projection matrix $W_{g}\in R^{2K\times K}$. The fused representation is computed as:


(4) $$X_{h}=\sigma (W_{g}[F_{\text{ESM}};F_{\text{DCC}}])⊙(F_{\text{ESM}}+F_{\text{DCC}}),$$

where σ scales gating weights to [0, 1] for adaptive feature weighting. The concatenated features integrate global semantics and local patterns, while the gating matrix captures their interactions. This modulation emphasizes biologically relevant residues, such as active or mutation-prone sites.

### 2.3 Protein functional attention

In PF-AGCN, we introduce a novel attention mechanism—protein functional attention—designed to capture complex dependencies in protein–protein functional relationships. This framework comprises two synergistic modules: “protein attention,” which learns intra-protein dependencies and interaction patterns, and “function attention,” which models semantic relationships between functional categories. Together, these components enable effective learning of cross-level dependencies essential for protein function understanding.

The “function attention” module exploits the hierarchical structure of GO domains, which encode latent functional dependencies among GO terms. To model these correlations, we define a functional correlation matrix $F=[f_{j,j^{\prime }}]\in R^{N_{F}\times N_{F}}$, computed as:


(5) $$F=V_{f}\cdot \sigma \left(\left(X_{h}^{r-1}U_{1}\right)U_{2}\left(U_{3}X_{h}^{r-1}\right)+b_{f}\right),$$

where $X_{h}^{r-1}\in R^{N_{F}\times N_{P}\times C_{r-1}}$ represents hidden features from the previous layer, and $U_{1}$, $U_{2}$, $U_{3}$, $V_{f}$, and $b_{f}$ are learnable parameters. Each entry $f_{j,j^{\prime }}$ quantifies the semantic association between functions *j* and $j^{\prime }$. We apply sparse normalization to retain only the most informative relations:


(6) $${\tilde{f}}_{j,j^{\prime }}=\frac{\;\text{exp}(f_{j,j^{\prime }})\cdot I(f_{j,j^{\prime }}\in \mathrm{TopK}(f_{j,j^{\prime }},\lambda ))}{\sum_{k\in N_{F}}\;\text{exp}\;(f_{j,k})\cdot I(f_{j,k}\in \;\mathrm{TopK}(f_{j,k},\lambda ))},$$

where I(·) denotes the indicator function, returning 1 if the condition is true and 0 otherwise. λ is a learnable TopK ratio, and TopK selects the top λ correlations for each function node. To address the rigidity of fixed TopK ratios, which limit adaptability to varying data and model needs, we introduce a dynamic λ that adjusts based on data characteristics, enhancing model flexibility and performance.

The “protein attention” module learns pairwise protein relevance without requiring predefined interaction networks, enabling end-to-end learning of protein dependencies from raw input. Given hidden protein representations $X_{h}^{r-1}\in R^{N_{P}\times N_{P}\times C_{r-1}}$, we compute the protein correlation matrix $P=[p_{i,i^{\prime }}]\in R^{N_{P}\times N_{P}}$ as:


(7) $$P=V_{p}\cdot \sigma \left(\left(X_{h}^{r-1}W_{1}\right)W_{2}{\left(W_{3}X_{h}^{r-1}\right)}^{T}+b_{p}\right),$$

with learnable weights $W_{1}$, $W_{2}$, $W_{3}$, $V_{p}$, and $b_{p}$. To enhance interpretability and reduce noise, we normalize P via sparse attention:


(8) $${\tilde{p}}_{i,i^{\prime }}=\frac{\;\text{exp}(p_{i,i^{\prime }})\cdot I(p_{i,i^{\prime }}\in \mathrm{TopK}(p_{i,i^{\prime }},\lambda ))}{\sum_{k\in N_{i}}\;\text{exp}\;(p_{i,k})\cdot I(p_{i,k}\in \mathrm{TopK}(p_{i,i^{\prime }},\lambda ))},$$

where $k=\left\lfloor N_{P}/4\right\rfloor$ determines the sparsity level, ensuring the model focuses on the most functionally relevant protein–protein and function–function relationships, thereby improving performance in downstream protein function prediction tasks.

### 2.4 Adaptive graph convolution

Accurate protein function prediction requires modeling complex conditional dependencies (Yang *et al.* 2020). We propose an adaptive graph convolutional framework that jointly learns both the “function graph” and “protein graph” structures via end-to-end training, incorporating two key modules: AF-GCB and AP-GCB. Unlike conventional GAT-based methods, our approach compensates for incomplete prior knowledge in predefined adjacency matrices while preserving native graph structures, enhancing biological interpretability. In GO graphs, relations like “is_a” and “part_of” are inherently unidirectional (Yang *et al.* 2023), and unidirectional diffusion better preserves graph heterogeneity and minimizes signal degradation (Perrault-Joncas and Meila 2011). Conversely, PPI networks involve bidirectional interactions, where bidirectional diffusion captures mutual information flow more effectively, enriching feature propagation and improving prediction of protein interactions (Li *et al.* 2019).

The self-adaptive function adjacency matrix $\tilde{F}$, derived from Eq. (6), captures directed relationships among protein functions. To embed prior domain knowledge, we incorporate a GO-based category tree via an adjacency matrix $A_{F}$, which is fused with $\tilde{F}$ to modulate inter-node influence dynamically. Recognizing the directed nature of functional relationships, we implement a unidirectional diffusion convolution layer over *N* steps. Given the function attention matrix ${\tilde{F}}_{n}\in R^{N_{F}\times N_{F}}$ at step *n*, the function graph convolution is defined as:


(9) $$Z_{F}=\sum_{n=1}^{N}A_{F}^{n}X_{F}W_{n,1}+{\tilde{F_{n}}}^{n}X_{F}W_{n,2},$$

where $X_{F}\in R^{N_{F}\times N_{P}\times C_{\text{in}}}$ is the input feature tensor and $Z_{F}\in R^{N_{F}\times N_{F}}$ is the output. $W_{n,1}$ and $W_{n,2}\in R^{C_{\text{in}}\times C_{\text{out}}}$ are learnable parameters. Without prior adjacency, the adaptive function graph convolution simplifies to:


(10) $$Z_{F}=\sum_{n=1}^{N}{\tilde{F}}^{n}X_{F}W_{n,2}.$$

To construct the protein graph, we first use BLAST to measure protein sequence similarity, producing a binary adjacency matrix $A_{P}\in R^{N_{P}\times N_{P}}$. A connection is established between proteins *j* and $j^{\prime }$ if their *E*-value, $B_{j,j^{\prime }}$, is below a predefined threshold *E*:


(11) $$A_{P}(j,j^{\prime })=\{\begin{array}{cc} 1, & \text{if}\;B_{j,j^{\prime }}<E,\\ 0, & \text{otherwise}. \end{array}$$

While BLAST can identify homologous sequences, it does not imply functional interaction. In PF-AGCN, we construct a sequence similarity network (SSN) via BLAST alignment, using it as an initial approximation of the PPI network. The true PPI structure is then refined through an attention mechanism. To capture latent dependencies, we introduce a self-adaptive protein adjacency matrix $\tilde{P}$ (see Eq. (8)). Given the dynamic and reciprocal nature of PPIs, we model the protein graph as bidirectional.

The protein graph convolution employs a bidirectional diffusion mechanism over *N* steps:


(12) $$Z_{P}=\sum_{n=1}^{N}R_{f}^{n}X_{P}U_{n,1}+R_{b}^{n}X_{P}U_{n,2}+{\tilde{P}}^{n}X_{P}U_{n,3},$$

where $X_{P}\in R^{N_{F}\times N_{P}\times C_{\text{in}}}$ is the input, $Z_{P}\in R^{N_{F}\times N_{P}\times C_{\text{in}}}$ is the output, and $U_{n,1}$, $U_{n,2}$, $U_{n,3}\in R^{E_{\text{in}}\times E_{\text{out}}}$ are learnable weights. The normalized forward and backward diffusion matrices, including self-loops, are defined as:


(13) $$\{\begin{array}{c} R_{f,n}=A_{P,n}/\text{rowsum}(A_{P,n}),\\ R_{b,n}={\left(A_{P,n}\right)}^{T}/\text{rowsum}\left({\left(A_{P,n}\right)}^{T}\right). \end{array}$$

If no predefined adjacency is used, the adaptive protein graph convolution simplifies to:


(14) $$Z_{P}=\sum_{n=1}^{N}{\tilde{P}}^{n}X_{P}U_{n,3}.$$

### 2.5 Loss function

We use Binary Cross-Entropy (BCE) with logits loss to quantify the discrepancy between predicted logits and binary ground truth labels in multi-label classification. This loss is well-suited for such tasks, treating each label independently and applying a sigmoid activation to map logits to probabilities. The model is trained to minimize this loss by aligning predicted probabilities with true labels across all functional categories. The BCE with logits loss is formally defined as:


(15) $$\begin{array}{cc} & \text{Loss}\left(Y,\hat{Y}\right)=\\ & \frac{1}{N_{P}}\sum_{j\in N_{P}}\left[\max(0,Y_{j})-Y_{i}{\hat{Y}}_{j}+\text{log}\;\left(1+\text{exp}\;\left(1-\left|Y_{j}\right|\right)\right)\right], \end{array}$$

where $Y_{j}$ denotes the model’s prediction (logit) and ${\hat{Y}}_{j}$ the corresponding binary ground truth label for the *j*th protein.

## 3 Experiments

### 3.1 Datasets

We adopt GO as the functional annotation framework following the CAFA protocol. The GO dataset, sourced from http://geneontology.org/page/download-ontology, contains 44 683 classes (excluding 1968 obsolete ones) organized into three ontologies. Due to computational constraints and sparse annotations for highly specific terms, we apply an annotation frequency-based ranking strategy (Kulmanov *et al.* 2018). Our dataset, derived from UniProtKB and compliant with CAFA standards, includes protein sequences and their GO annotations, with detailed statistics in Table 1. Each ontology-specific dataset comprises eight components: accessions, GO terms, labels, n-grams, protein IDs, sequences, organisms, and embeddings. UniProtKB serves as the primary data source, providing comprehensive annotations, sequence data, taxonomy, protein names, literature references, and curated metadata. For evaluation, we randomly sampled 10 000 proteins of varying lengths from diverse organisms and used PF-AGCN to predict functions across all three GO domains.

**Table 1.** Specific information about the three ontologies in the database.

Full name	Abbr.	Proteins	Functions
Molecular function	MF	10 161	589
Biological process	BP	28 647	932
Cellular component	CC	390	436

### 3.2 Experimental configuration and settings

Experiments were conducted using an NVIDIA GeForce RTX 4090 with NVIDIA-SMI driver version 528.24 and CUDA 11.7. A grid search determined the optimal batch size of 128, and all models were trained for 50 epochs. To maintain an 8:2 training-to-test split, we employed a diffusion convolutional layer with a default propagation step N=2. Separate models were trained for each GO, using dilation rates of 2, 4, and 8 in the DCC module. The learning rate was set to 0.001, dropout to 20%, and the random seed to 42. Each experiment was repeated five times to ensure robustness against random variation. The ESM-1b model was used as a frozen feature extractor, while all other layers were trained with full-precision parameters. Training the complete model required approximately 4.58 h.

Model performance was evaluated using standard CAFA metrics, including precision, recall, maximum *F*-score ($F_{\text{max}}$), and area under the receiver operating characteristic (ROC) curve (AUC), ensuring comparability with previous works (Radivojac *et al.* 2013, Kulmanov *et al.* 2018, Zhou *et al.* 2019). $F_{\text{max}}$ offers a robust measure of classifier performance across multiple categories and is defined as:

(16) $$F_{\text{max}}=\underset{h}{\max}\frac{2\times \bar{\mathrm{Pre}(h)}\times \bar{Re(h)}}{\bar{\mathrm{Pre}(h)}+\bar{Re(h)}},$$

where $\bar{\mathrm{Pre}(h)}$ and $\bar{Re(h)}$ are the average precision and the average recall of all proteins at threshold *h*, respectively.

Let $V_{F}=\{g_{1},g_{2},\ldots ,g_{i},\ldots ,g_{N_{F}}\}$ represent GO terms. For the *j*th protein, precision and recall at threshold *h* are calculated as:

(17) $$Pre_{j}(h)=\frac{\sum_{i\in N_{F}}I(g_{i}\in P_{j}(h)\wedge g_{i}\in T_{j})}{\sum_{i\in N_{F}}I(g_{i}\in P_{j}(h))},$$

 

(18) $$Re_{j}(h)=\frac{\sum_{i\in N_{F}}I(g_{i}\in P_{j}(h)\wedge g_{i}\in T_{j})}{\sum_{i\in N_{F}}I(g_{i}\in T_{j})},$$

where $P_{j}(h)$ and $T_{j}$ are the predicted and true GO term sets for protein *j*, and I(·) is an indicator function.

The average precision and recall across all $N_{P}$ proteins are:

(19) $$\bar{\mathrm{Pre}(h)}=\frac{1}{N_{P}}\sum_{j\in N_{P}}Pre_{j}(h),\;\bar{Re(h)}=\frac{1}{N_{P}}\sum_{j\in N_{P}}Re_{j}(h).$$

The AUC quantifies the model’s ability to distinguish between classes and is computed as:

(20) $$\text{AUC}=\int_{-\infty }^{\infty }\frac{TP(h)}{TP(h)+FN(h)}\left(\frac{-FP(h)}{FP(h)+TN(h)}\right)dh,$$

where TP(h),TN(h),FP(h),FN(h) represent true positives, true negatives, false positives, and false negatives at threshold *h*, respectively.

### 3.3 Baselines

We benchmarked PF-AGCN against several representative protein function prediction models:

- BLAST (Cozzetto *et al.* 2016): A sequence similarity-based method that transfers annotations from homologous proteins in the training set to test samples.
- DeepLSTM (Hashemifar *et al.* 2018): A deep LSTM network that extracts complex sequential features from protein sequences.
- DeepGo (Kulmanov *et al.* 2018): Combines CNN-based sequence encoding with a cross-species PPI network.
- PANDA2 (Zhao *et al.* 2022): Utilizes a graph neural network to model GO DAG structure, integrating features from transformer-based protein language models.
- GAT-GO (Lai and Xu 2022): Employs GATs to integrate predicted structural and sequence embeddings for function prediction.
- NetGo 3.0 (Wang *et al.* 2023a): Uses ESM-1b-derived protein embeddings as input to a logistic regression model for GO term association.
- DeepGO-SE (Kulmanov *et al.* 2024): Applies a pre-trained large language model (ESM-2) to predict GO terms from protein sequences.

### 3.4 Ablation studies

To assess the contribution of individual components in PF-AGCN, we conducted ablation experiments on key modules, including AP-GCB, AF-GCB, and adaptive graph learning:

- P-AGCN: Removes AF-GCB, retaining only AP-GCB and the output layer to capture PPI features via protein graph convolution.
- F-AGCN: Removes AP-GCB, focusing solely on functional information from the GO graph.
- PF-GAT: Replaces graph convolution with attention-based updates, as defined in Equations (10) and (14).
- PF-noDCC: Removes the DCC module, relying solely on ESM for sequence features, thus eliminating the modeling of local dependencies.
- PF-noGM: Replaces the gating mechanism with simple concatenation to integrate global semantics and local patterns.

This comparative analysis highlights the relative strengths and weaknesses of PF-AGCN, offering insights into the impact of each architectural component. The subsequent subsection discusses experimental results in detail.

## 4 Results and analysis

**Figure 3.**
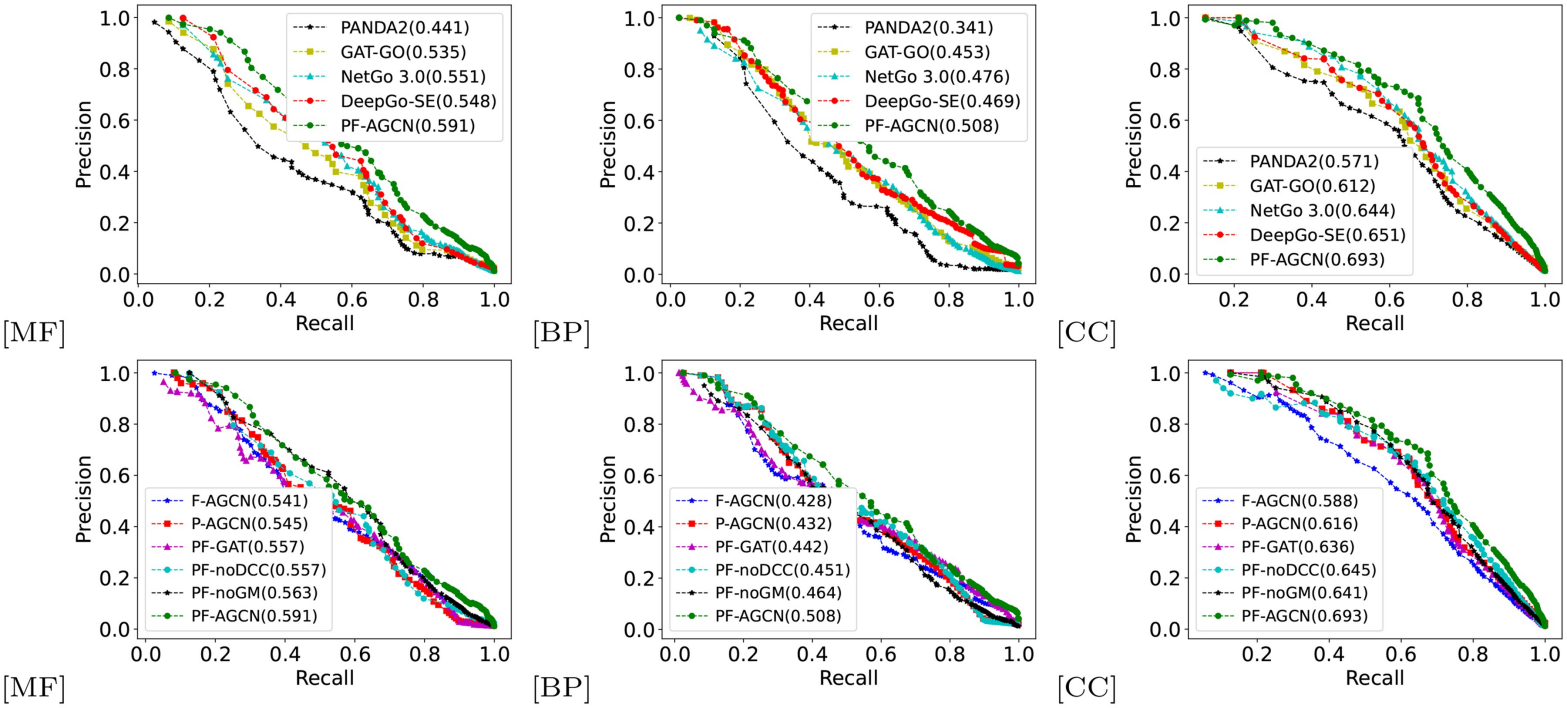
Precision–recall curves on GO terms across three ontologies: (a–c) baseline models (PANDA2, GAT-GO, NetGo 3.0, DeepGo-SE, PF-AGCN) on MF, BP, and CC; (d–f) ablation models (F-AGCN, P-AGCN, PF-GAT, PF-noDCC, PF-noGM, PF-AGCN) on the same datasets. $F_{\text{max}}$ scores are annotated in the legends.

As shown in Fig. 3a–c and Table 2, PF-AGCN consistently outperforms baselines, with average gains of 126.3% over BLAST, 18.7% over DeepGO, 26.9% over PANDA2, 8.2% over DeepGO-SE, 12.4% over GAT-GO, and 7.3% over NetGO 3.0. Paired *t*-tests confirm that these improvements are statistically significant (*P* < .05) across all datasets.

**Table 2.** Prediction results of the baselines.

Method	MF				BP				CC			
	$F_{\text{max}}$	$\bar{Pre(h)}$	$\bar{Re(h)}$	AUC	$F_{\text{max}}$	$\bar{Pre(h)}$	$\bar{Re(h)}$	AUC	$F_{\text{max}}$	$\bar{Pre(h)}$	$\bar{Re(h)}$	AUC
BLAST	0.274 ± 0.000	0.266 ± 0.000	0.282 ± 0.000	**–**	0.211 ± 0.000	0.173 ± 0.000	0.271 ± 0.000	**–**	0.232 ± 0.000	0.215 ± 0.000	0.251 ± 0.000	**–**
DeepGo	0.319 ± 0.000	0.319 ± 0.009	0.319 ± ± 0.008	0.628 ± 0.002	0.282 ± 0.002	0.308 ± 0.009	0.287±0.019	0.477 ± 0.002	0.585 ± 0.002	0.5277 ± 0.008	0.581 ± 0.019	0.757 ± 0.002
PANDA2	0.441 ± 0.083	0.432 ± 0.105	0.430 ± 0.033	0.681 ± 0.003	0.341 ± 0.001	0.398 ± 0.011	0.393 ± 0.013	0.552 ± 0.000	0.571 ± 0.002	0.587 ± 0.011	0.573 ± 0.010	0.715 ± 0.000
GAT-GO	0.535 ± 0.001	0.531 ± 0.036	0.532 ± 0.033	0.781 ± 0.002	0.453 ± 0.003	0.447 ± 0.089	0.439 ± 0.101	0.615 ± 0.000	0.612 ± 0.006	0.632 ± 0.011	0.629 ± 0.026	0.834 ± 0.008
DeepGO-SE	0.548 ± 0.002	0.536 ± 0.006	0.547 ± 0.001	0.819 ± 0.003	0.469 ± 0.005	0.451 ± 0.048	0.465 ± 0.092	0.676 ± 0.001	0.651 ± 0.010	0.641 ± 0.008	0.664 ± 0.035	0.881 ± 0.009
NetGo 3.0	0.551 ± 0.001	0.541 ± 0.036	0.552 ± 0.021	0.821 ± 0.002	0.476 ± 0.002	0.453 ± 0.080	0.471 ± 0.102	0.670 ± 0.000	0.644 ± 0.006	0.633 ± 0.011	0.671 ± 0.026	0.875 ± 0.005
**PF-AGCN**	**0.591** ± **0.002**	**0.568** ± **0.000**	**0.560** ± **0.005**	**0.872** ± **0.001**	**0.508** ± **0.001**	**0.484** ± **0.003**	**0.485** ± **0.004**	**0.706** ± **0.000**	**0.693** ± **0.000**	**0.672** ± **0.002**	**0.684** ± **0.000**	**0.935** ± **0.001**

Bold values indicate the performance of the proposed method.

As shown in Fig. 3d–f and Table 3, F-AGCN resulted in the largest drop in $F_{\text{max}}$ for the MF task (Δ=−0.041), highlighting the importance of protein graph convolution for modeling PPI features. Conversely, P-AGCN showed the largest AUC decline for BP, emphasizing the role of functional graph convolution in enhancing classification confidence. PF-noGM exhibited consistently lower performance across all GO domains, confirming the effectiveness of the gating mechanism in integrating global semantics and local structural patterns. PF-AGCN also incorporates an attention mechanism specifically designed for biological networks, accounting for their sparsity, directionality, and interpretability. The performance gap between PF-AGCN and PF-GAT across all tasks supports this design; in MF, PF-GAT’s 2.5% lower $F_{\text{max}}$ stems from its inability to model GO term directionality (e.g. “binding”→“catalytic activity”), while in BP, its 3.3% AUC drop reflects noise from non-informative dense edges. These findings show that naïvely applying standard GATs to biological graphs can degrade performance. Finally, PF-noDCC caused a 0.025 reduction in $F_{\text{max}}$ and a 0.007 drop in AUC for MF, indicating that while the DCC module enhances recall and AUC, its effect on precision is limited. Overall, AF-GCB emerges as the most impactful component of PF-AGCN.

**Table 3.** Prediction results of the ablation models.

Method	MF				BP				CC			
	$F_{\text{max}}$	$\bar{Pre(h)}$	$\bar{Re(h)}$	AUC	$F_{\text{max}}$	$\bar{Pre(h)}$	$\bar{Re(h)}$	AUC	$F_{\text{max}}$	$\bar{Pre(h)}$	$\bar{Re(h)}$	AUC
P-AGCN	0.545 ± 0.001	0.547 ± 0.036	0.541 ± 0.033	0.812 ± 0.002	0.432 ± 0.003	0.430 ± 0.089	0.457 ± 0.007	0.634 ± 0.000	0.616 ± 0.006	0.623 ± 0.011	0.622 ± 0.026	0.889 ± 0.008
F-AGCN	0.541 ± 0.083	0.548 ± 0.105	0.540 ± 0.033	0.842 ± 0.006	0.428 ± 0.001	0.416 ± 0.012	0.433 ± 0.013	0.621 ± 0.000	0.588 ± 0.002	0.596 ± 0.013	0.593 ± 0.010	0.915 ± 0.000
PF-GAT	0.557 ± 0.001	0.556 ± 0.036	0.546 ± 0.033	0.859 ± 0.002	0.442 ± 0.003	0.456 ± 0.080	0.465 ± 0.092	0.676 ± 0.000	0.636 ± 0.006	0.627 ± 0.011	0.619 ± 0.026	0.902 ± 0.008
PF-noDCC	0.557 ± 0.001	0.562 ± 0.009	0.555 ± 0.041	0.865 ± 0.005	0.451 ± 0.004	0.464 ± 0.080	0.469 ± 0.062	0.687 ± 0.000	0.645 ± 0.008	0.637 ± 0.016	0.628 ± 0.043	0.914 ± 0.014
PF-noGM	0.563 ± 0.007	0.560 ± 0.002	0.551 ± 0.001	0.862 ± 0.035	0.464 ± 0.024	0.468 ± 0.052	0.464 ± 0.025	0.683 ± 0.004	0.641 ± 0.025	0.635 ± 0.042	0.664 ± 0.016	0.907 ± 0.005
**PF-AGCN**	**0.591** ± **0.002**	**0.568** ± **0.000**	**0.560** ± **0.005**	**0.872** ± **0.001**	**0.508** ± **0.001**	**0.484** ± **0.003**	**0.485** ± **0.004**	**0.706** ± **0.000**	**0.693** ± **0.000**	**0.672** ± **0.002**	**0.684** ± **0.000**	**0.935** ± **0.001**

Bold values indicate the performance of the proposed method.

**Figure 4.**
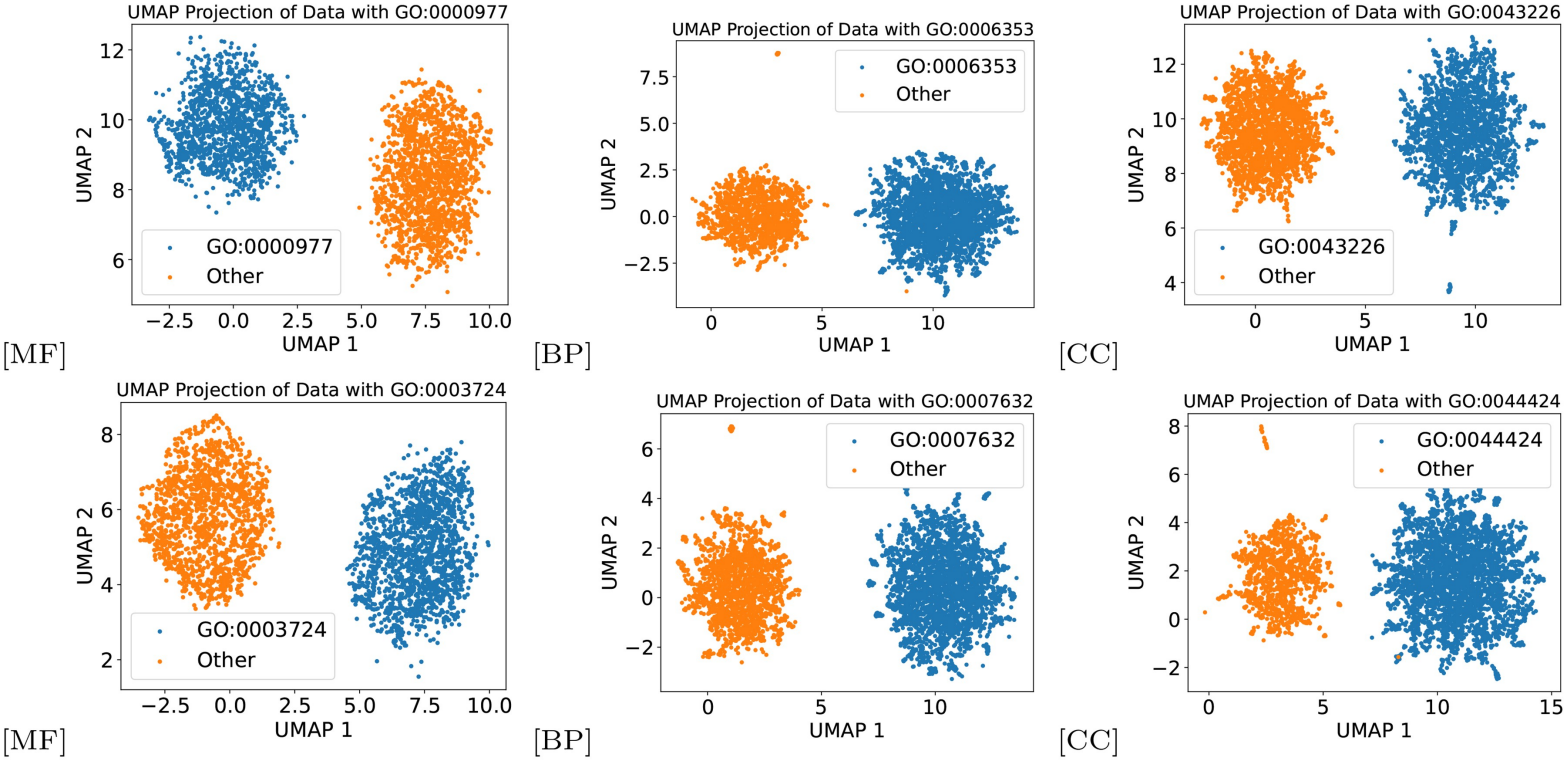
UMAP projections of test set embeddings illustrating functional hierarchy within each GO ontology. For MF, BP, and CC, the embeddings of the two largest GO categories are visualized using UMAP.

**Figure 5.**
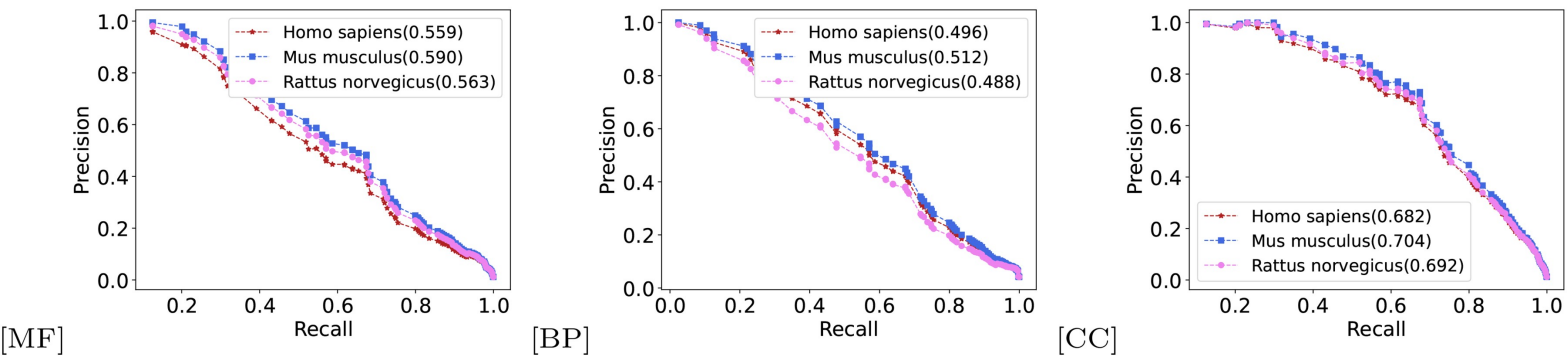
Precision–recall curves comparing PF-AGCN performance across different organisms, with $F_{\text{max}}$ annotated in the legends.

AP-GCB and AF-GCB jointly capture sequence- and graph-based protein features by modeling structural and semantic relationships at the protein and functional levels. The resulting embeddings, visualized via uniform manifold approximation and projection (UMAP) (Fig. 4), effectively separate GO domains despite visualization complexity, supporting the validity of the model architecture. As shown in Fig. 5, PF-AGCN achieves consistent performance across species, with the highest accuracy in *Homo sapiens*, demonstrating strong generalizability across diverse biological contexts.

To evaluate the impact of BLAST E-value thresholds on PPI edge construction, we performed a sensitivity analysis, as shown in Table 4. While lower thresholds such as $10^{-10}$ reduce noise, they may also exclude valid interactions (Couto *et al.* 2008). Starting from a permissive threshold of $10^{-5}$, we progressively tightened the criterion to assess its effect on prediction performance. The results show that an *E*-value of $10^{-8}$ provides the optimal balance, yielding the best performance for SSN construction.

**Table 4.** Prediction results under different *E*-value thresholds.

E-value	MF				BP				CC			
	$F_{\text{max}}$	$\bar{Pre(h)}$	$\bar{Re(h)}$	AUC	$F_{\text{max}}$	$\bar{Pre(h)}$	$\bar{Re(h)}$	AUC	$F_{\text{max}}$	$\bar{Pre(h)}$	$\bar{Re(h)}$	AUC
$10^{-5}$	0.552 ± 0.003	0.548 ± 0.030	0.556 ± 0.020	0.876 ± 0.022	0.471 ± 0.002	0.459 ± 0.060	0.471 ± 0.102	0.670 ± 0.000	0.654 ± 0.006	0.653 ± 0.061	0.671 ± 0.026	0.875 ± 0.005
$10^{-8}$	0.574 ± 0.003	0.557 ± 0.020	0.546 ± 0.008	0.857 ± 0.004	0.501 ± 0.007	0.463 ± 0.030	0.470 ± 0.083	0.690 ± 0.000	0.675 ± 0.001	0.676 ± 0.011	0.676 ± 0.006	0.918 ± 0.024
$10^{-10}$	**0.591** ± **0.002**	**0.568** ± **0.000**	**0.560** ± **0.005**	**0.872** ± **0.001**	**0.508** ± **0.001**	**0.484** ± **0.003**	**0.485** ± **0.004**	**0.706** ± **0.000**	**0.693** ± **0.000**	**0.672** ± **0.002**	**0.684** ± **0.000**	**0.935** ± **0.001**
$10^{-15}$	0.577 ± 0.002	0.554 ± 0.030	0.545 ± 0.002	0.862 ± 0.035	0.493 ± 0.008	0.477 ± 0.012	0.473 ± 0.002	0.691 ± 0.003	0.691 ± 0.002	0.662 ± 0.000	0.672 ± 0.027	0.920 ± 0.005

Bold values indicate results with an E-value of 10^−10^.

**Figure 6.**
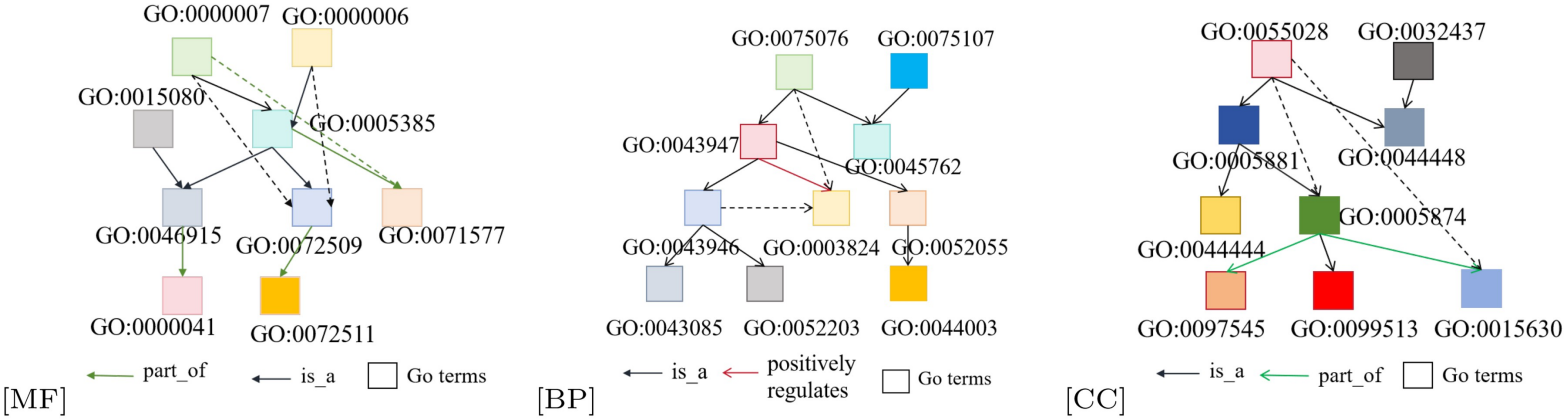
DAG graphs of GO terms in MF, BP, and CC derived from the proposed attention mechanism. Solid lines indicate retained true edges; dashed lines denote filtered ones.

**Figure 7.**
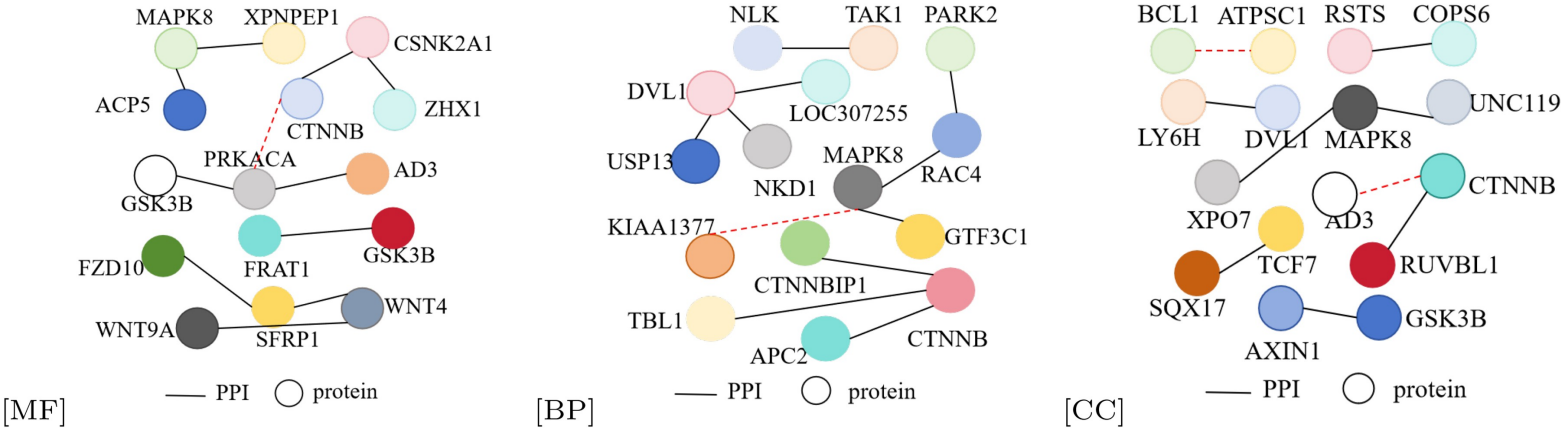
PPI results from the proposed functional attention across MF, BP, and CC are visualized using randomly selected proteins. Solid lines denote correctly predicted PPIs, while dashed lines indicate incorrect ones.

We conducted visualization-based comparisons between the original GO DAG and protein contact graphs and those derived from the proposed protein and function attention mechanisms. As shown in Fig. 6, the protein functional attention effectively removes redundant GO relations (e.g. secondary “is_a” links), though some valid connections—such as GO:0043946 and GO:0003824 in BP—are occasionally omitted. Figure 7 shows an 11:1 ratio of correct to incorrect PPI predictions, suggesting some deviation from native PPI structures. Nevertheless, PF-AGCN significantly outperforms baselines, validating its capability in modeling PPI and GO semantics.

## 5 Conclusion and future studies

This article presents PF-AGCN, an adaptive GCN for protein function prediction. PF-AGCN integrates the ESM transformer encoder to capture long-range residue dependencies and evolutionary patterns, while using DCC to extract local features like secondary structure motifs and hydrophobic regions. To model functional associations, we propose a protein functional attention mechanism that combines “function attention” for inter-category relations and “protein attention” for protein–protein dependencies. The model consists of two core modules: AF-GCB and AP-GCB, which fuse attention with adaptive GCN layers to enhance representation learning. These components enable PF-AGCN to capture protein graph features while preserving structural context. Experiments show that PF-AGCN consistently outperforms baselines, with ablation studies validating the roles of AF-GCB and AP-GCB in leveraging PPI data.

Cross-species evaluations confirm its robustness and generalizability. In sparse PPI networks, limited connectivity may impair information flow. To mitigate this, future work will incorporate biological priors or multi-omics data (e.g. gene expression) to enrich sparse regions. We also plan to explore hybrid models combining topological and multi-omics inputs, and to develop scalable algorithms with regularization techniques to address computational challenges in large ontologies.
